# Supplementary material for: Twisted photonic Weyl meta-crystals and aperiodic Fermi arc scattering
Source: Nat Commun. 2024 Mar 18;15:2440. doi: 10.1038/s41467-024-46759-x (PMC10948390; doi:10.1038/s41467-024-46759-x)
Supplement: Supplementary file 1 — Supplementary Information [file 41467_2024_46759_MOESM1_ESM.pdf]

SUPPLEMENTARY INFORMATION for "Twisted photonic Weyl meta-crystals and aperiodic Fermi arc scattering"

Hanyu Wang, Wei Xu, Zeyong Wei, Yiyuan Wang, Zhanshan Wang, Xinbin Cheng, Qinghua Guo, Jinhui Shi, Zhihong Zhu, Biao Yang

Contents

I. Effective medium of Weyl metamaterials 2
II. Reflection matrix 3
III. Arcless Fermi loops and influence of trivial propagating phase 5
IV. Extended RCWA and round-trip eigen phase 7
V. Transmission from RCWA 11
VI. Experimental sample 13
VII. Experimental results 14
VIII. Fermi arcs arising from scattering 15
References 16

I. Effective medium of Weyl metamaterials

We start from effective media theory, the constitutive relation is given as,

$$\begin{pmatrix}\mathbf{D}\\ \mathbf{B}\end{pmatrix}=\begin{pmatrix}\overleftrightarrow{\epsilon} & \overleftrightarrow{\zeta}\\ \overleftrightarrow{\xi} & \overleftrightarrow{\mu}\end{pmatrix}\begin{pmatrix}\mathbf{E}\\ \mathbf{H}\end{pmatrix}=\begin{pmatrix}\overleftrightarrow{\epsilon_r}\,\epsilon_0 & \frac{i}{c}\overleftrightarrow{\chi}\\ -\frac{i}{c}\overleftrightarrow{\chi}^T & \overleftrightarrow{\mu_r}\,\mu_0\end{pmatrix}\begin{pmatrix}\mathbf{E}\\ \mathbf{H}\end{pmatrix} \qquad (S1)$$

where the sign convention is $\mathbf{E}(\mathbf{r},t)=\mathbf{E}_0e^{i(\omega t-\mathbf{k}\cdot\mathbf{r})}$. For the top Weyl metamaterial (fixed), we have,

$$\overleftrightarrow{\epsilon_r}=\begin{pmatrix}\epsilon_m & 0 & 0\\ 0 & \epsilon_m & 0\\ 0 & 0 & \epsilon_z\end{pmatrix},\overleftrightarrow{\mu_r}=\begin{pmatrix}\mu_m & 0 & 0\\ 0 & \mu_m & 0\\ 0 & 0 & \mu_z\end{pmatrix},\overleftrightarrow{\chi}=\begin{pmatrix}0 & \chi_m & 0\\ \chi_m & 0 & 0\\ 0 & 0 & 0\end{pmatrix} \qquad (S2)$$

where for simplicity, we already assume $\epsilon_0=\mu_0=c=1$ and $l=1, A=0.9, L=1, \omega_0=1$. Components in the constitutive relation is given as $\epsilon_m=1+\frac{l^2}{L}\frac{1}{\omega_0^2-\omega^2}$, $\mu_m=1+\frac{A^2}{L}\frac{\omega^2}{\omega_0^2-\omega^2}$, $\chi_m=\frac{lA}{L}\frac{\omega}{\omega_0^2-\omega^2}$, and $\epsilon_z=\mu_z=1$.

The Weyl nodes derived from Eq. S1 consist of a transverse mode and a longitude mode[1], where the crossing of two modes forms Weyl nodes. The nonlocal effect, resulting from inter-unit-cell interaction, bends the longitudinal mode to be dispersive with negative group velocity, and slightly moves the Weyl nodes. Due to the nonlocal effect, l is replaced by $l_n=1-\frac{\alpha(k_x^2+k_y^2)}{\beta+\gamma(k_x^2+k_y^2)}$, where $\alpha=1, \beta=10, \gamma=1$. Thus, ϵ_m and χ_m are given as $\epsilon_m=1+\frac{l_n^2}{L}\frac{1}{\omega_0^2-\omega^2}$ and $\chi_m=\frac{l_nA}{L}\frac{\omega}{\omega_0^2-\omega^2}$, respectively. Then the twisted Fermi arcs (see Fig. 1b) from the effective medium perspective are shown in Supplementary Figure 1.

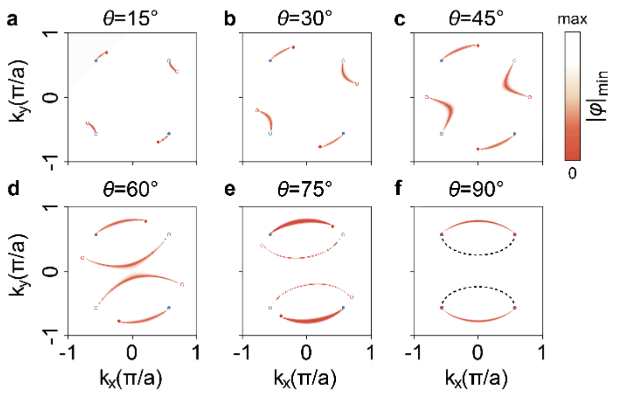

Supplementary Figure 1. Twisted Fermi arcs in the twisted bi-block Weyl metamaterials.

II. Reflection matrix

The reflection of the bottom meta-crystal is discussed first. We need two modes to form the basis for electromagnetic waves. Here we use $(|Down_1\rangle, |Down_2\rangle)$ to represent all possible basis,

$$|Down_1\rangle = [E_{1x}, E_{1y}, E_{1z}, H_{1x}, H_{1y}, H_{1z}]^T \qquad (S3a)$$

$$|Down_2\rangle = [E_{2x}, E_{2y}, E_{2z}, H_{2x}, H_{2y}, H_{2z}]^T \qquad (S3b)$$

Note that the state space of incident and reflected basis are different. We introduce a mirror operator $\widehat{M}$ to connect them,

$$|Down_1\rangle = \widehat{M}|Up_1\rangle, |Down_2\rangle = \widehat{M}|Up_2\rangle \qquad (S4)$$

The matrix representation of $\widehat{M}$ is,

$$\widehat{M} = \begin{pmatrix} 1 & 0 & 0 & 0 & 0 & 0 \\ 0 & 1 & 0 & 0 & 0 & 0 \\ 0 & 0 & -1 & 0 & 0 & 0 \\ 0 & 0 & 0 & -1 & 0 & 0 \\ 0 & 0 & 0 & 0 & -1 & 0 \\ 0 & 0 & 0 & 0 & 0 & 1 \end{pmatrix} \qquad (S5)$$

For simplicity, we choose $(|TE_{down}\rangle, |TM_{down}\rangle)$ as the basis for downward incidence,

and take $\hat{R}$ as the reflection operator, the reflection matrix is constructed by,

$$\begin{cases} \hat{R}|TE_{down}\rangle = a|TE_{up}\rangle + b|TM_{up}\rangle \\ \hat{R}|TM_{down}\rangle = c|TE_{up}\rangle + d|TM_{up}\rangle \end{cases} \quad (S6)$$

Further applying mirror $\hat{M}$ to connect the two basis, the matrix representation of $\hat{R}\hat{M}$ is $\hat{R}\hat{M}|i\rangle = \sum_j D_{ji}|j\rangle$. Then, we obtain the reflection matrix as,

$$D(\hat{R}\hat{M}) = \begin{pmatrix} a & c \\ b & d \end{pmatrix} \quad (S7)$$

Note that the reflection matrix is the representation of $\hat{R}\hat{M}$ rather than $\hat{R}$. For a total reflection, the reflection matrix $D(\hat{R}\hat{M})$ is a unitary matrix.

Next, we consider a wave propagating along a round-trip, i.e., experiencing twice reflections. Note that for the round-trip, we do not need to connect two basis state spaces, that is, the state space is always the same after the round-trip. We define $\hat{R}'$ as the reflection operator of the top meta-crystal,

$$\begin{cases} \hat{R}'|TE_{up}\rangle = a'|TE_{down}\rangle + b'|TM_{down}\rangle \\ \hat{R}'|TM_{up}\rangle = c'|TE_{down}\rangle + d'|TM_{down}\rangle \end{cases} \quad (S8)$$

Finally, we have the matrix representation by following $\hat{R}'\hat{R}|i\rangle = \sum_j D_{ji}|j\rangle$,

$$D(\hat{R}'\hat{R}) = \begin{pmatrix} aa' + bc' & ca' + dc' \\ ab' + bd' & cb' + dd' \end{pmatrix} = \begin{pmatrix} a' & c' \\ b' & d' \end{pmatrix}\begin{pmatrix} a & c \\ b & d \end{pmatrix} \quad (S9)$$

$$D(\hat{R}'\hat{R}) = D(\hat{R}'\hat{M})D(\hat{R}\hat{M}) \quad (S10)$$

The round-trip reflection matrix describes the two reflection processes, which can be calculated separately and combined simply using matrix multiplication.

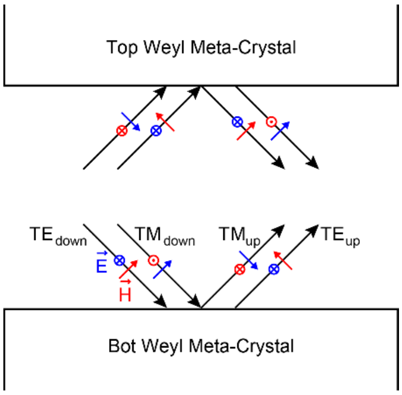

Supplementary Figure 2. Relation between basis in two state spaces, i.e., $(|TE_{down}\rangle, |TM_{down}\rangle)$ and $(|TE_{up}\rangle, |TM_{up}\rangle)$.

III. Arcless Fermi loops and influence of trivial propagating phase

In Figs. 2d and e, we show the round-trip eigen phases φ varying from $-\pi \to \pi$ when the path encircled one Weyl point, there must be a single zero point that is $\varphi = 2n\pi$. Therefore, the relation is always satisfied when $\varphi^{II} = 0$. When $\varphi^{II} \neq 0$ the relation could be still satisfied as long as φ^{II} is topologically trivial. Usually, φ^{II} is introduced by the trivial spacer layer, that means no singularity (unlike φ^{I}). Increasing h leads to cavity mode appearing in the dielectric spacer layer, those isolated trivial Fermi loops are detached from bulk states, which are mentioned in Ref. [2,3] as arcless surface states as shown in Supplementary Figure 3a. When the twisting angle $\theta \neq 0$ in Supplementary Figures 3b and c, different values of h introduce a trivial extra phase φ^{II} upon the Riemann surface phase φ, which leads to various Fermi arcs. Notably, the top and bottom Weyl meta-crystals could be considered independently when h is large enough, since the two meta-crystals are fully decoupled.

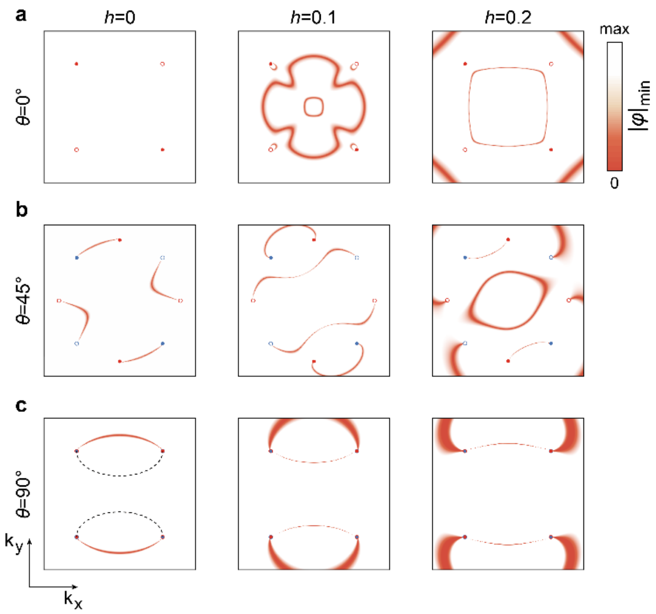

Supplementary Figure 3. Fermi arcs varying with different spacer layer thicknesses h when $\theta = 0$, 45° and 90°, corresponding to a, b and c, respectively. The dielectric constant of spacer layer $\epsilon = 16$.

We show several φ^{II} with changing the thickness as shown in Supplementary Figures 4a-d, one sees there is no any singularity. Accordingly, Fermi arcs varies as shown in Supplementary Figures 4e-g, with the corresponding round trip eigen phases shown in Supplementary Figures 4h-j. In a word, different φ^{II} just rotates Fermi arc state around each Weyl point while the relation $\varphi = \varphi^{I} + \varphi^{II} = 2n\pi$ is always satisfied.

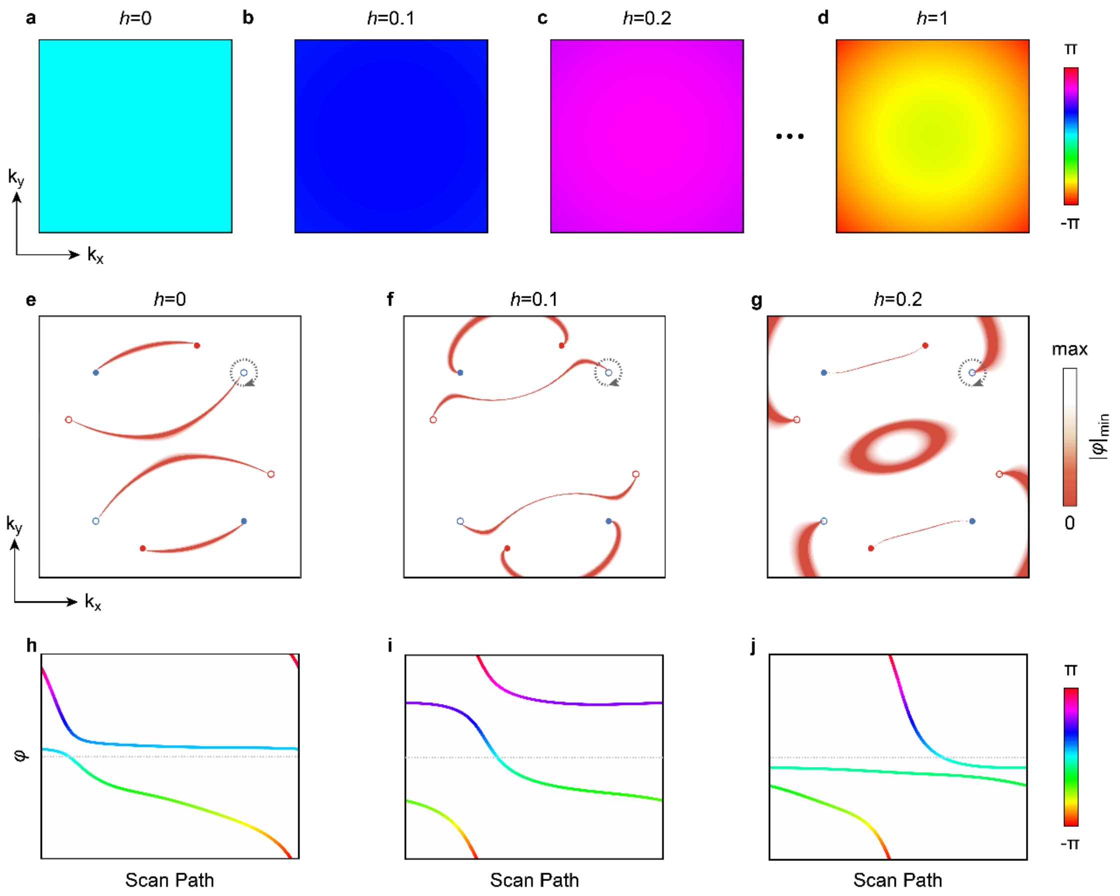

Supplementary Figure 4. Fermi arc changing with trivial phase φ^{II}. a-d, Different φ^{II} corresponding to different dielectric spacer layer thickness h with the dielectric constant $\epsilon = 16$. **e-g**, Fermi arcs change for different h, the twisting angle $\theta = 60°$. **h-j**, Round-trip eigen phases around Weyl point, where the scan paths are indicated in (e-g). The horizontal dashed lines denote the guided mode condition $\varphi = \varphi^{I} + \varphi^{II} = 2n\pi$.

IV. Extended RCWA and round-trip eigen phase

Extended RCWA with effective media theory

We combine rigorous coupled wave analysis (RCWA) with effective media theory to calculate the twisted Fermi arcs considering periodic modulation. Due to nonlocal

effect, we assume that the entry ϵ_m is related to the in-plane momentum (k_x, k_y). Thus, we only introduce refractive index modulation on ϵ_z. RCWA method requires Fourier decomposition of the dielectric function $\epsilon_z = \sum_{\mathbf{g}\in G} \bar{\epsilon}_z(\mathbf{g}) \cdot e^{i\mathbf{g}\cdot\mathbf{r}}$, and $\mathbf{r}$ is the in-plane coordinate in real space, and $\mathbf{g} \in G$ are the reciprocal lattice vectors. Similarly expanding the electromagnetic field as,

$$\mathbf{E}_\beta(x,y,z) = \sum_{\mathbf{g}\in G} S_\beta(\mathbf{g},z) e^{-i(k_x(\mathbf{g})x + k_y(\mathbf{g})y)} \quad (S11a)$$

$$\mathbf{H}_\beta(x,y,z) = \sum_{\mathbf{g}\in G} U_\beta(\mathbf{g},z) e^{-i(k_x(\mathbf{g})x + k_y(\mathbf{g})y)} \quad (S11b)$$

where $\beta \in (x,y,z)$ are the fields components, $\mathbf{k}_\beta(\mathbf{g}) = \mathbf{k}_{inc,\beta} + \mathbf{g}_\beta$ for incident plane wave wavevector $\mathbf{k}_{inc}$. The existence of electromagnetic coupling term $\overleftrightarrow{\chi}$ makes coupled equations a more complex form than the conventional RCWA method[4,5]. The matrix form of Maxwell's equation is written as:

$$-i\tilde{k}_y S_z - \frac{d}{d\tilde{z}} S_y = [\mu_m]U_x - [\chi_m]S_y \quad (S12a)$$

$$\frac{d}{d\tilde{z}} S_x + i\tilde{k}_x S_z = [\mu_m]U_y - [\chi_m]S_x \quad (S12b)$$

$$-i\tilde{k}_x S_y + i\tilde{k}_y S_x = [\mu_z]U_z \quad (S12c)$$

and,

$$-i\tilde{k}_y U_z - \frac{d}{d\tilde{z}} U_y = [\epsilon_m]S_x - [\chi_m]U_y \quad (S13a)$$

$$\frac{d}{d\tilde{z}} U_x + i\tilde{k}_x U_z = [\epsilon_m]S_y - [\chi_m]U_x \quad (S13b)$$

$$-i\tilde{k}_x U_y + i\tilde{k}_y U_x = [\epsilon_z]S_z \quad (S13c)$$

We eliminate k_0 with $\tilde{k}_i = \frac{k_i}{k_0}$, $\tilde{z} = k_0 z$, where $\tilde{k}_i$ is a diagonal matrix, and use $[\epsilon_z]$, $[\mu_z]$ and $[\chi_m]$ to denote the convolution matrices of the ϵ_z, μ_z, and χ_m, respectively.

The longitudinal field component is given as,

$$S_z = i[\epsilon_z]^{-1}\left(\tilde{k}_y U_x - \tilde{k}_x U_y\right) \tag{S14a}$$

$$U_z = i[\mu_z]^{-1}\left(\tilde{k}_y S_x - \tilde{k}_x S_y\right) \tag{S14b}$$

Finally, the coupled equations are,

$$\frac{d}{d\tilde{z}}\begin{pmatrix} S_x \\ S_y \\ U_x \\ U_y \end{pmatrix} = \begin{pmatrix} \overleftrightarrow{X} & \overleftrightarrow{P} \\ \overleftrightarrow{Q} & \overleftrightarrow{X} \end{pmatrix}\begin{pmatrix} S_x \\ S_y \\ U_x \\ U_y \end{pmatrix} \tag{S15}$$

where,

$$\overleftrightarrow{P} = \begin{pmatrix} \tilde{k}_x[\epsilon_z]^{-1}\tilde{k}_y & [\mu_m] - \tilde{k}_x[\epsilon_z]^{-1}\tilde{k}_x \\ \tilde{k}_y[\epsilon_z]^{-1}\tilde{k}_y - [\mu_m] & -\tilde{k}_y[\epsilon_z]^{-1}\tilde{k}_x \end{pmatrix} \tag{S16}$$

$$\overleftrightarrow{Q} = \begin{pmatrix} \tilde{k}_x[\mu_z]^{-1}\tilde{k}_y & [\epsilon_m] - \tilde{k}_x[\mu_z]^{-1}\tilde{k}_x \\ \tilde{k}_y[\mu_z]^{-1}\tilde{k}_y - [\epsilon_m] & -\tilde{k}_y[\mu_z]^{-1}\tilde{k}_x \end{pmatrix} \tag{S17}$$

$$\overleftrightarrow{X} = \begin{pmatrix} -[\chi_m] & 0 \\ 0 & [\chi_m] \end{pmatrix} \tag{S18}$$

Since the bottom Weyl meta-crystal is twisted by an angle θ, the constitutive matrices are,

$$\overleftrightarrow{\epsilon_r}(\theta) = J\overleftrightarrow{\epsilon_r}J^{-1} = \begin{pmatrix} \epsilon_m & 0 & 0 \\ 0 & \epsilon_m & 0 \\ 0 & 0 & \epsilon_z \end{pmatrix} \tag{S19a}$$

$$\overleftrightarrow{\mu_r}(\theta) = J\overleftrightarrow{\mu_r}J^{-1} = \begin{pmatrix} \mu_m & 0 & 0 \\ 0 & \mu_m & 0 \\ 0 & 0 & \mu_z \end{pmatrix} \tag{S19b}$$

$$\overleftrightarrow{\chi}(\theta) = J\overleftrightarrow{\chi}J^{-1} = \begin{pmatrix} sin(2\theta)\,\chi_m & cos(2\theta)\,\chi_m & 0 \\ cos(2\theta)\,\chi_m & -sin(2\theta)\,\chi_m & 0 \\ 0 & 0 & 0 \end{pmatrix} \tag{S19c}$$

where,

$$J = \begin{pmatrix} cos(\theta) & sin(\theta) & 0 \\ -sin(\theta) & cos(\theta) & 0 \\ 0 & 0 & 1 \end{pmatrix} \tag{S20}$$

The coupled equation for the bottom meta-crystal could be constructed following the

above process in a similar way, and θ is the twisted angle.

$$\frac{d}{d\tilde{z}}\begin{pmatrix} S_x \\ S_y \\ U_x \\ U_y \end{pmatrix} = \begin{pmatrix} \overleftrightarrow{X}(\theta) & \overleftrightarrow{P}(\theta) \\ \overleftrightarrow{Q}(\theta) & \overleftrightarrow{X}(\theta) \end{pmatrix}\begin{pmatrix} S_x \\ S_y \\ U_x \\ U_y \end{pmatrix} \tag{S21}$$

where,

$$\overleftrightarrow{P}(\theta) = \begin{pmatrix} \tilde{k}_x[\epsilon_z]^{-1}\tilde{k}_y & [\mu_m] - \tilde{k}_x[\epsilon_z]^{-1}\tilde{k}_x \\ \tilde{k}_y[\epsilon_z]^{-1}\tilde{k}_y - [\mu_m] & -\tilde{k}_y[\epsilon_z]^{-1}\tilde{k}_x \end{pmatrix} \tag{S22}$$

$$\overleftrightarrow{Q}(\theta) = \begin{pmatrix} \tilde{k}_x[\mu_z]^{-1}\tilde{k}_y & [\epsilon_m] - \tilde{k}_x[\mu_z]^{-1}\tilde{k}_x \\ \tilde{k}_y[\mu_z]^{-1}\tilde{k}_y - [\epsilon_m] & -\tilde{k}_y[\mu_z]^{-1}\tilde{k}_x \end{pmatrix} \tag{S23}$$

$$\overleftrightarrow{X}(\theta) = \begin{pmatrix} -\,cos(2\theta)\,[\chi_m] & sin(2\theta)[\chi_m] \\ sin(2\theta)\,[\chi_m] & cos(2\theta)\,[\chi_m] \end{pmatrix} \tag{S24}$$

The extended RCWA method considers all the reciprocal lattices $(\mathbf{k}_{inc} + \mathbf{g}^{(1)} + \mathbf{g}^{(2)} | \mathbf{g}^{(1)} \in G_1, \mathbf{g}^{(2)} \in G_2)$, the number of elements in G_i is infinite theoretically. One always neglects the high-order plane waves, i.e., beyond a certain threshold. With the extended RCWA, one can easily get the transmission, reflection, and absorption of the twisted bi-block Weyl meta-crystals.

Round-trip eigen phase

Here, in order to obtain the twisted Fermi arcs, we want the round-trip eigen phases of the spacer cavity between the two twisted Weyl meta-crystals. The top and bottom blocks are totally reflected, and a semi-infinite condition is used to ensure that the surface states decay upwards and downwards, respectively. The thickness of spacer layer is tunable and can be zero as a critical condition.

The general form of scattering matrix can be constructed as,

$$\begin{pmatrix} c_1^- \\ c_2^+ \end{pmatrix} = \begin{pmatrix} \tilde{S}_{11} & \tilde{S}_{12} \\ \tilde{S}_{21} & \tilde{S}_{22} \end{pmatrix} \begin{pmatrix} c_1^+ \\ c_2^- \end{pmatrix} \tag{S25}$$

We combine the top and bottom scattering matrices, aiming to construct a scattering matrix characterizing round-trip eigen mode, which is the twisted Fermi arcs. We define $\tilde{S}^{\mathrm{top}}$ as the scattering matrix describing reflection on the interface of L_1 and L_2, providing the connection between the up-propagating modes and down-propagating modes on the top surface is $c_2^+ = \tilde{S}_{22}^{\mathrm{top}} c_2^-$. Similarly, the reflection on the interface of L_3 and L_4 providing $c_3^- = \tilde{S}_{11}^{\mathrm{bot}} c_3^+$. When the thickness of spacer layer is 0, we have $c_3^- = c_2^-$, and $c_2^+ = \tilde{S}_{22}^{\mathrm{top}} c_2^- = \tilde{S}_{22}^{\mathrm{top}} \tilde{S}_{11}^{\mathrm{bot}} c_3^+$. For the round-trip reflection operator $\hat{R}|i\rangle = \sum_j D_{ji}|j\rangle$, we have the matrix representation as $D(\hat{R}) = \tilde{S}_{22}^{\mathrm{top}} \tilde{S}_{11}^{\mathrm{bot}}$. For total reflections, the reflection matrix $D(\hat{R})$ is a unitary matrix, whose eigenvalues take the form of $e^{i\varphi_m}$ with φ_m indicating the round-trip eigen phases. The general process is similar to this one, e.g., considering a spacer layer with $h \neq 0$, where matching the boundary conditions across each interface is required.

V. Transmission from RCWA

The scattering energy is not uniformly distributed for all orders. Usually, the low order contains the maximum scattering, while other orders are relatively weak, leading to only several Fermi arcs remaining bright in the near-field scanning experiment. Figure

S5a shows that only a sparse selection of Fermi arcs is lightened during the transmission process with the Direct Product basis. Compared with Supplementary Figure 5a, Supplementary Figure 5b shows that the transmission result of Direct Sum is much more concise after eliminating other higher order basis and is acceptable to map the bright twisted Fermi arcs.

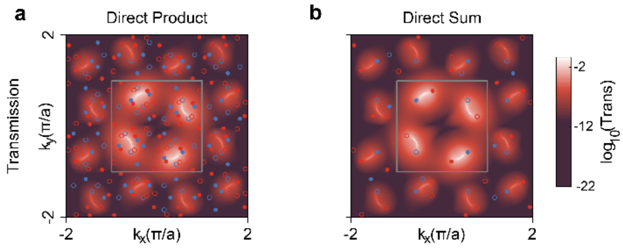

Supplementary Figure 5. Transmission spectrum of RCP (right circular polarization) incident light obtained from Direct Product (a) and Direct Sum (b), respectively. The bright areas represent to Fermi arcs, which correspond to transmission peaks.

VI. Experimental sample

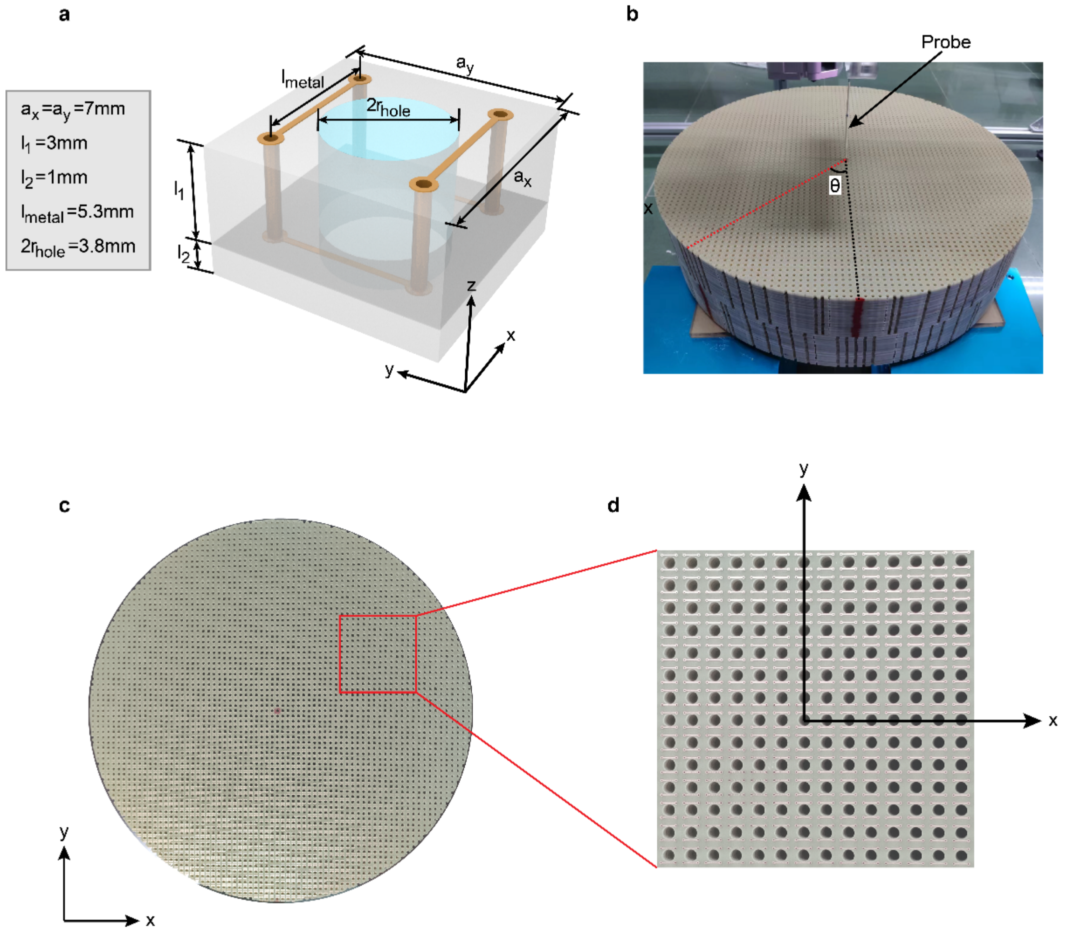

Supplementary Figure 6. Details of the experimental sample. a, Dimension parameters of Weyl meta-crystal used in the experiment. The size of plated through holes depends on the specific printed circuit board (PCB) technology and will not influence the results. The copper thickness is 1oz (35μm). **b,** The top and bottom Weyl meta-crystals are relatively twisted by an angle θ. **c,** Top view of the sample. The central red point indicates the center hole, where the source antenna inserted from the bottom block. There are 71 unit-cells along both x and y directions (i.e. its diameter has

71 unit-cells). **d,** The zoom-in view. The probe antenna raster-scans the periodic holes (see details in Method).

VII. Experimental results

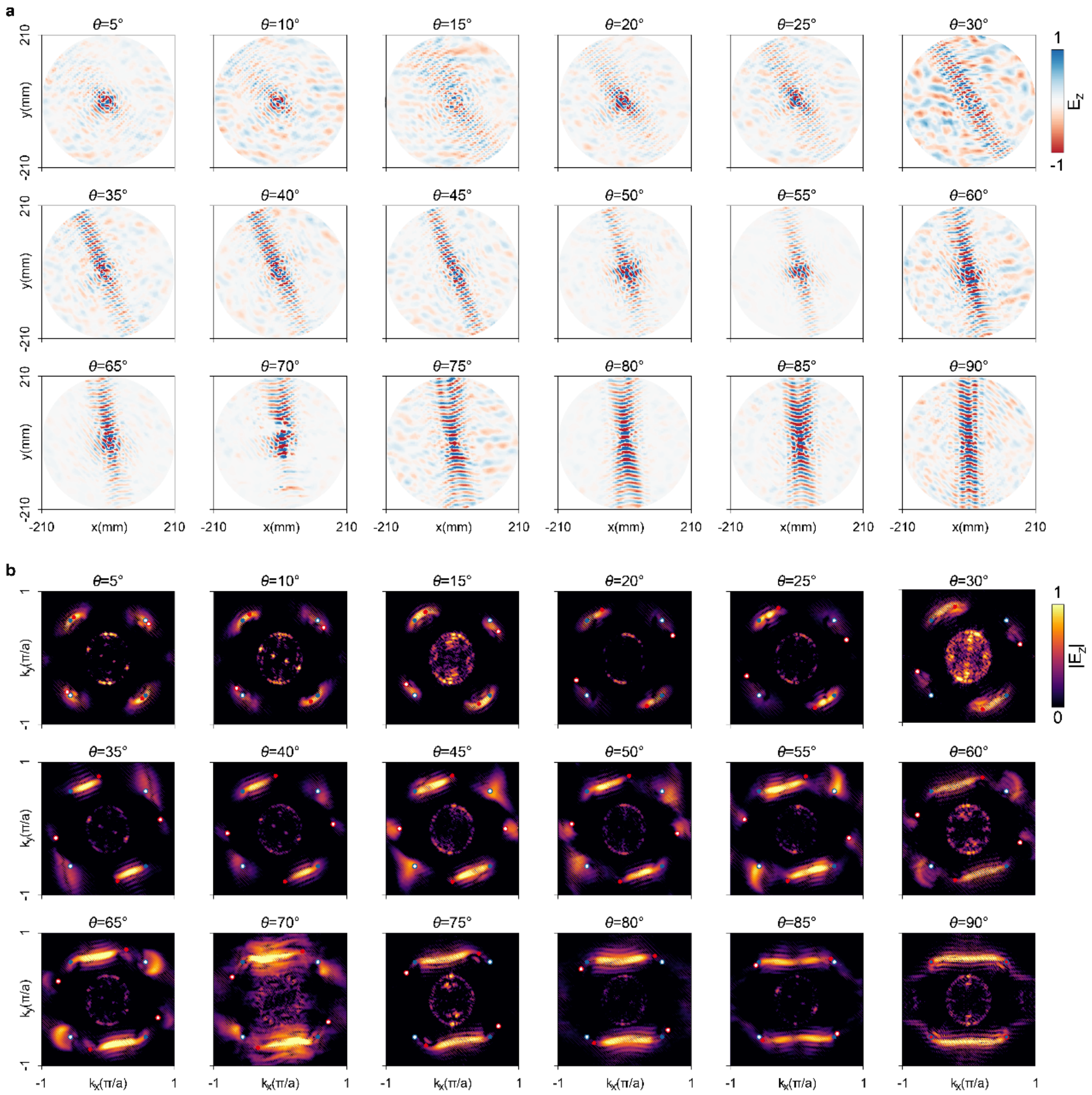

Supplementary Figure 7. Experiment results of continuously twisted Fermi arcs. **a**, Real space pattern. **b**, Fermi arcs in momentum space after Fourier transformation.

VIII. Fermi arcs arising from scattering

In effective media theory, there are only four Weyl points for each Weyl block as shown in Fig. 1a. Considering the weak periodic modulation, more Weyl points are introduced. Specifically, N plane waves introduce to $4N$ Weyl points, that is, effective media theory counts only the zeroth plane wave. For small twisting angles θ, the Fermi arcs appearing in the top FBZ are dominated by the zeroth plane wave. With increasing the twisting angle θ, Fermi arcs tend to bridge high-order Weyl points arising from scattering and reconstruct across the top FBZ, as shown in Supplementary Figures 8a and b.

As indicated by the dashed arrow in Supplementary Figure 8a, Fermi arc runs across the top FBZ, bridging another Weyl point outside. With increasing frequency, the Fermi arc rotates clockwise and gets close to another Fermi arc. The corresponding experimental mapping of Fermi arc evolution is another strong support of our RCWA method. In Supplementary Figure 8a, we manipulate the connectivity of Fermi arcs by shift the trivial phase φ^{II}. As previously mentioned, the adjustment of the phase φ^{II}, variation of the twist angle, and modification of frequencies are viable approaches for reconstructing Fermi arcs.

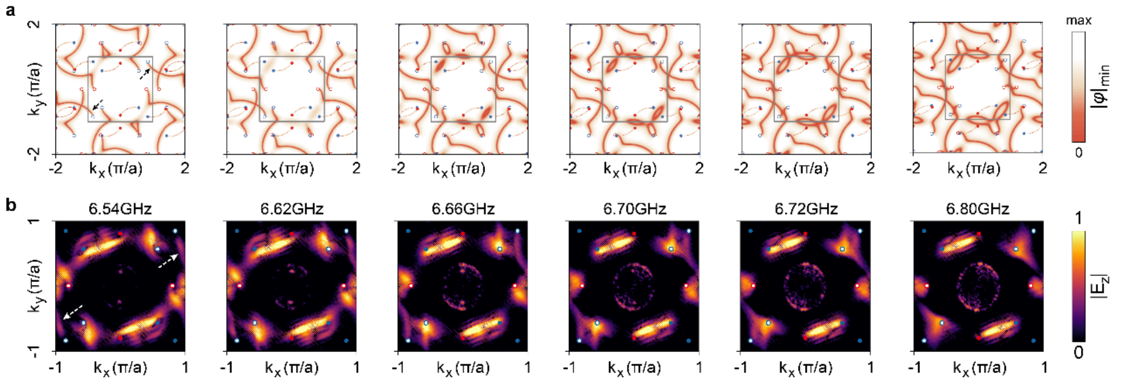

Supplementary Figure 8. Fermi arcs bridging high order Weyl points. a, Fermi arcs obtained from Direct Sum of RCWA when the twisting angle $\theta = 45°$. **b**, Experimentally mapped Fermi arcs corresponding to panel **a**.

References

1 Yang, B. *et al.* Ideal Weyl points and helicoid surface states in artificial photonic crystal structures. *Science* **359**, 1013-1016, doi:10.1126/science.aaq1221 (2018).

2 Murthy, G., Fertig, H. A. & Shimshoni, E. Surface states and arcless angles in twisted Weyl semimetals. *Physical Review Research* **2**, 013367, doi:10.1103/PhysRevResearch.2.013367 (2020).

3 Dwivedi, V. Fermi arc reconstruction at junctions between Weyl semimetals. *Physical Review B* **97**, 064201, doi:10.1103/PhysRevB.97.064201 (2018).

4 Lou, B. *et al.* Theory for Twisted Bilayer Photonic Crystal Slabs. *Physical Review Letters* **126**, 136101, doi:10.1103/PhysRevLett.126.136101 (2021).

5 Lou, B., Wang, B., Rodríguez, J. A., Cappelli, M. & Fan, S. Tunable guided resonance in twisted bilayer photonic crystal. *Science Advances* **8**, eadd4339, doi:10.1126/sciadv.add4339 (2022).
